# Significance of norovirus in occupational health: a review of published norovirus outbreaks in Central and Northern Europe

**DOI:** 10.1007/s00420-020-01543-4

**Published:** 2020-05-01

**Authors:** Felix Martin Hofmann, Edward Olawumi, Martina Michaelis, Ulrich Stößel, Friedrich Hofmann

**Affiliations:** 1 Research Centre for Occupational and Social Medicine (FFAS), Bertoldstraße 63, 79098 Freiburg, Germany; 2 Institute of Earth and Environmental Sciences, University of Freiburg, Albertstraße 23b, 79104 Freiburg, Germany

**Keywords:** Central/Northern Europe, Norovirus gastroenteritis, Occupational health, Outbreak

## Abstract

**Objectives:**

Globally, norovirus (NoV) is the leading cause of gastroenteritis infection among all ages. The development of prevention strategies in the field of occupational health requires a detailed knowledge about the impact of the disease on employees. This review article aims not only at evaluating the burden of NoV outbreaks on staff but also at discussing implications for future prevention strategies.

**Methods:**

Published NoV outbreaks in Central and Northern Europe were identified via a systematic literature search. Additionally, published NoV outbreaks in Germany were detected via a manual literature search. Key epidemiological data, as the number of symptomatic staff, was then extracted. The proportion of affected employees was calculated for each dataset (single NoV outbreaks or aggregated data of multiple outbreaks).

**Results:**

Overall, 116 datasets were extracted from 72 relevant articles. 144,852 persons were affected by NoV gastroenteritis, 25,408 out of them (17.5%) were employees. 23,874 (94.0%) of them fell sick during outbreaks in hospitals and related settings. NoV cases among personnel in food establishments were reported only sporadically (mean ratio: 0.01).

**Conclusions:**

Employees in hospitals and community facilities seem quantitatively to be most vulnerable towards NoV epidemics. Therefore, high quality of prevention measures in these settings, respective compliance with prevention strategies should have the highest priority. The disease can be considered as an occupational disease, even regularly without long-term consequences. Following work safety rules, a vaccination for vulnerable groups should be recommended if the vaccine development turns out to be successful.

**Electronic supplementary material:**

The online version of this article (10.1007/s00420-020-01543-4) contains supplementary material, which is available to authorized users.

## Introduction

Norovirus (NoV) infection is the leading cause of sporadic disease and outbreaks of acute gastroenteritis worldwide (Glass et al. 2009; Lopman et al. 2015). The prevalence of NoV is highest in developing countries (Lanata et al. 2013). The extremely contagious virus affects individuals of all ages, but young children and the elderly remain the most vulnerable groups (Glass et al. 2009). An annual incidence rate in England as high as 34 consultations/1000 person-years in children younger than five years has been reported, thereby confirming the unequivocal vulnerability of this group to NoV infections (Verstraeten et al. 2017).

The infection usually lasts only a few days and subsides without any lasting damage, but symptoms, such as diarrhoea, vomiting, nausea, stomach pain, headache, and in certain cases also fever, may persist longer in young children, elderly and in immunocompromised individuals (Roddie et al. 2009), whereby a NoV infection may be fatal for the latter group (Schwartz et al. 2011). Humans remain the only known reservoir for transmission, which occurs mostly oral-faecal (e.g. by contact of the hands with contaminated areas) and vomit-oral (by the oral intake of droplets containing the virus): “This explains the very rapid spread of infection within nursing homes, hospitals and community facilities” (RKI 2019a). Direct person-to-person transmission, food, water and environmental fomites have been identified as the predominant transmission routes. Since the infection only occurs in humans, the virus is ultimately transmitted from person-to-person (Lopman et al. 2015). Noroviruses of genogroup (GG) II have been the predominant strains within recent years (Pringle et al. 2015). GG 2 genotype 4 (GGII.4) strains seem to be associated with significantly higher hospitalisation and mortality rates (Desai et al. 2012). It was demonstrated in an early study that NoV infected individuals develop a temporary immunity of about 6–14 weeks after a contact with the virus (Parrino et al. 1977), whereas a recent study points towards a longer protection that may last for several years (Simmons et al. 2013). Several factors, such as an incomplete understanding of the immunity against NoV hamper current efforts aiming at developing a vaccine against NoV (Atmar et al. 2011; Riddle and Walker 2016; Cortes-Penfield et al. 2017; Lucero et al. 2018).

Despite the visibly high burden of NoV worldwide, it is worth noting that official statistics are not representative. Indeed, a considerable underestimation of the true incidence must be assumed due to a disproportion between symptomatic persons, laboratory testing and notification of cases to official data registers (Tam et al. 2012; Bernard et al. 2014c). In Germany, for example, NoV gastroenteritis is a notifiable disease since 2001 according to the Protection against Infection Act (Infektionsschutzgesetz) (RKI 2019a). A notification to the federal public health authority is mandatory in the case of at least two affected persons and causal epidemic assumptions, in the case of at least one affected person working in the food processing sector, and for children under 6 years who acquired the infection in childcare institutions. Nationwide surveillance data is available in an electronic database at the Robert Koch Institute (RKI), the public health institute on disease surveillance, control and prevention (Bernard et al. 2014b). Considering that only laboratory-confirmed cases have to be reported to the RKI since 2011 (RKI 2011), it raises the question as to whether later statistics of the RKI are representative. In an overview of the number of cases of notifiable diseases in 2017, the RKI itself acknowledges that their reported number of NoV cases may be misleading (RKI 2018).

Between the end of 2013 and January 2015, the number of officially registered NoV gastroenteritis cases decreased, but still remained the most frequently reported disease amounting to about 75,040 laboratory-confirmed cases with an incidence rate of 92.9 per 100,000 persons in 2014 (RKI 2016). A somewhat similar number of cases (*n* = 73,273) was reported to the RKI in 2017 (RKI 2018). In addition to the problems associated with the reporting systems, stool samples of outpatient cases of acute gastroenteritis are not routinely tested for NoV (Schmutz et al. 2017; Hofmann et al. 2020a, b).

NoV outbreaks are ubiquitous but most prevalent in healthcare and community facilities, thereby resulting in significant morbidity and substantial healthcare costs (Pringle et al. 2015). Forty-five percent, 29% and 11% of the NoV outbreaks in Germany occurred in health care facilities, nursing homes and childcare facilities, respectively (Höhne and Askar 2014). NoV outbreaks on cruise ships, for example, are frequently associated with both significant revenue losses and high numbers of symptomatic persons (Isakbaeva et al. 2005). It has been shown that NoV outbreaks on cruise ships may even affect three-quarters of the embarked passengers (Bert et al. 2014).

During NoV outbreaks in health care facilities, staff members are often affected, leading to severe staff shortages, temporary closure of wards (Harris et al. 2014) and, ultimately, economic losses (Zingg et al. 2005). Individual studies indicate a heterogeneous pattern regarding the proportion of sick employees during NoV outbreaks. A review of more than 70 NoV outbreaks in five different hospitals in Germany showed, for example, that 24% of the 1432 persons with clinical symptoms were employees (Mattner et al. 2015). Other studies indicate that the number of affected health care workers (HCWs) during hospital outbreaks may even exceed the number of affected patients, thereby leading to a serious burden for staff (Meyer et al. 2004; Mattner et al. 2006; Sideroglou et al. 2014; Schulz-Stübner et al. 2016). The high proportion of sick employees in hospital outbreaks may be partly explained by the fact that a specialised hygiene team develops infection control measures in most German hospitals, whereas the health of personnel belongs to the scope of occupational health physicians. Secondly, HCWs are at higher risk due to environmental contamination (Lopman et al. 2012). As shown in an own recent study (Michaelis et al. 2017), the latter group receives usually very little information about the number of sick employees and is thus often not actively involved in the outbreak management. Hence, it is anticipated that the lack of cooperation between the occupational health physicians and the specialised hygiene team may lead to a higher number of cases among HCWs in hospitals.

As previously highlighted (Michaelis et al. 2017), the impact of NoV outbreaks on employees in different settings has not yet been evaluated on a broader quantitative scale. Therefore, this paper aims at answering the following two main questions:

- How large is the proportion of employees relative to the total number of sick persons in a large compilation of previously published NoV outbreaks?
- Are there any significant differences in the proportion of affected staff members depending on the setting of the outbreak?

To be able to assess the significance of NoV in occupational health and to discuss implications for future prevention strategies, this study aims at synthesising published epidemiological data from either single NoV outbreaks or aggregated register data. To ensure comparability, we restricted our analysis of NoV outbreaks to a list of countries in Central and Northern Europe that are characterised by the similar economic and hygienic situation (France, Switzerland, Liechtenstein, Austria, Germany, Luxembourg, Belgium, the Netherlands, the United Kingdom, Ireland, Denmark, Sweden, Norway, Iceland, Finland).

## Methods

For the identification of previously published outbreaks of NoV gastroenteritis, we first performed a systematic literature search using the search engines Embase^®^, Global Health, GMS, GMS meetings and PubMed, whereby a similar search strategy was developed to ensure comparability. Due to the similar search strategies, only the systematic search in Embase^®^ will be described hereinafter. A detailed description of the literature search in the different search engines is given in the supplementary material.

For the first step of the systematic search in Embase^®^, we attempted to identify all articles about NoV using the keywords ‘Norovirus’, ‘Norwalk-like virus’ and related terms. Since this review focuses on outbreaks, it was then assessed whether the articles contained at least one of the following keywords and related terms: ‘outbreak’, ‘epidemic’, ‘pandemic’ and ‘disease outbreak’. As we were expecting to detect articles that contain information about the impact of NoV outbreaks on staff members, the text words ‘Personnel’, ‘Occupational’, ‘Staff’ and ‘Health personnel’ as well as ‘Employee’, ‘Worker’ and related terms were included in the search strategy. Only epidemiological descriptions that were published between January 1st 2000 and 2016 were considered eligible for further analysis. All publications in languages other than English, French and German were discarded. Lastly, all articles listed in Medline were excluded to reduce the number of unnecessary duplicates, as all relevant articles listed in Medline were accessed through the systematic search in PubMed.

Additionally, a systematic literature search was performed using the Outbreak Database (Vonberg et al. 2011). Firstly, all articles that are listed under ‘Norovirus’ in the GE category (microorganism, genus) were accessed. It was then assessed whether the articles in question were published between 1 January 2000 and 31 December 2016. For the third step, we sought to identify published epidemiological descriptions of NoV outbreaks that contain information about the impact of the outbreaks on staff. Accordingly, the term ‘Personnel’ in the NOC category (Outbreak/Cases/Group) was included as a condition. Since we aimed mainly at identifying descriptions of NoV outbreaks in health care facilities, all articles were discarded that are not listed under ‘inpatient care (not ICU)’, ‘intensive care unit’, ‘nursing home’ in the FY category (Outbreak/Setting/Facility).

**Fig. 1:**
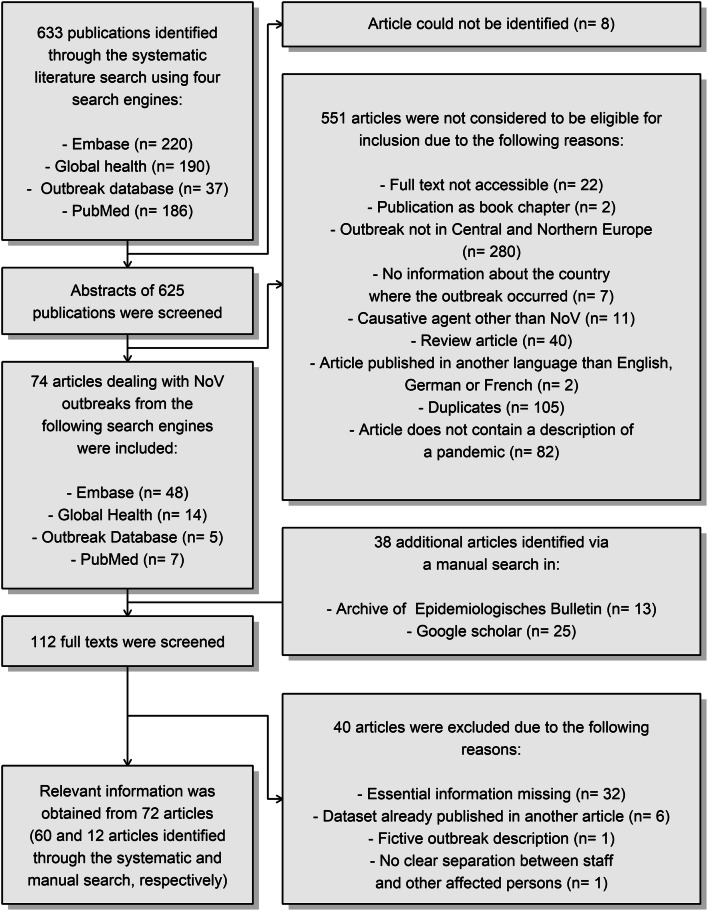
Flow chart of the systematic literature search and subsequent screening of relevant articles. The Fig. was created in RStudio 1.1.463

In total, 633 results were obtained during the systematic literature search, whereby 186, 190 and 220 relevant articles were identified through PubMed, Global Health and Embase^®^, respectively. The systematic search in the Outbreak Database resulted in the identification of 37 publications, whereas no relevant article was found in GMS and GMS meetings (Fig. 1).

For the next step, abstracts of all relevant publications were screened to assess their suitability for this study. Eight of the relevant publications could not be identified and were therefore discarded. Hence, only 625 abstracts of relevant publications were screened (see again Fig. 1). The full texts of 22 publications were not accessible and, therefore, the corresponding articles were excluded. As we decided to exclude book chapters from our analysis, two additional publications were discarded as well as two articles published in languages other than English, French and German. We decided to ignore 40 review articles among the results, as it would have been too time consuming to check whether published descriptions of single NoV outbreaks in a review article were already cited in another review article, thereby leading to duplicates and, consequently, a bias in our analysis. Indeed, we believe that that the original publications should have been detected through the systematic literature search.

Despite the fact that the term ‘outbreak’ and related terms were included in the search strategy, 82 articles were identified that did not contain any description of a NoV epidemic. Hence, these articles were excluded. Another 11 articles dealt with disease outbreaks caused by pathogens other than NoV. 280 articles about outbreaks of NoV gastroenteritis outside of Central and Northern Europe were excluded. As no information about the location of the outbreak was available in seven publications, we were unable to evaluate their suitability. Some publications were identified by up to four search engines. Thus, 105 duplicates were excluded, leaving 74 articles for further investigations.

As our main project tackled the epidemiological situation in Germany, we were interested in this review to study particularly the impact of NoV gastroenteritis outbreaks on employees in Germany. So, we performed an additional manual literature search in Google Scholar using the term ‘norovirus gastroenteritis Germany’. We identified 25 additional articles in this way. Secondly, the archive of *Epidemiologisches Bulletin* was accessed using the term ‘Norovirus’ to obtain further epidemiological descriptions of NoV gastroenteritis outbreaks in Germany, thereby identifying further 13 relevant articles.

For the next step, the full texts of 74 and 38 relevant articles that were identified through the systematic and manual literature search, respectively, were screened. 32 articles contained too little information about the NoV outbreaks and were thus discarded. For example, the number of sick personnel was not reported in 17 articles thereof. As the assessment of the impact of NoV outbreaks on staff is the main scope of this paper, these articles were considered particularly unsuitable for further analysis. Six articles containing outbreak descriptions that had already been published elsewhere were discarded. The description of a NoV outbreak in a German hospital (Ebner and Meyer 2007) was excluded, as it remained unclear whether the epidemic in the publication actually took place or if the authors aimed at illustrating the event of a NoV outbreak. In one article, no clear distinction was possible between symptomatic persons and staff.

Overall, key epidemiological data was extracted from 72 articles. The following information was retrieved from the original publications:

- Country where the outbreak occurred.
- Setting where the outbreak took place (e.g. restaurant). The articles were later grouped in four categories according to the setting: (a) health care facilities (hospitals and rehabilitation centres, dominantly, at least one hospital), (b) community facilities (e.g. nursing or residential home), (c) hotels, restaurants and canteens, (d) other food establishments (e.g. conference centres).
- Transmission path.
- Involved genogroup(s) and genotype(s).
- Infected collectives (e.g. patients or HCWs).
- Subjects with symptoms, i.e. the number of cases according to the case definition given by the authors. See the next paragraph for discussion.
- Affected employees with symptoms, i.e. the number of staff who fell sick during an outbreak.
- Affected subjects (non-staff) with symptoms.

We renounced to document the attack rate of infected staff, since it was rarely reported. The extracted number of symptomatic persons corresponds to the number of cases according to the case definition in the original publications. A case in a retrospective cohort study is usually defined as a subject who experienced the onset of specific clinical symptoms at a specific time (Loury et al. 2015). Although (1) vomiting in more than half of the sick persons, (2) an average incubation period between 24 and 48 h, (3) a mean duration of sickness between 12 and 60 h, (4) and the absence of bacterial pathogens in faecal samples have been proposed as epidemiological features of NoV outbreaks (Kaplan et al. 1982) and are nowadays widely accepted (Patel et al. 2009), there is no gold standard for the definition of a NoV case based upon specific clinical symptoms. For example, each person was considered a NoV case who experienced diarrhea and/or vomiting in a cohort study (Vo et al. 2016), whereas nausea, vomiting, diarrhea, abdominal cramping, fever (≥ 38 °C), muscle pain, headache or multiple of these symptoms were regarded as indicative for a NoV infection in another study (Johansson et al. 2002).

Due to this heterogeneity, it would have obviously been preferable to use a standardised case definition. However, such an attempt would have been hampered by the limited epidemiological information in the publications. Moreover, sick staff members were not always considered part of a cohort and only limited information was available about their clinical symptoms in many cases (Makary et al. 2009). As the lack of a common case definition should be regarded as one shortcoming of this study, we shed further light on this issue in the discussion. Regarding the classification of gastroenteritis outbreaks as NoV outbreaks, we also accepted the decision of the authors when laboratory evidence was missing, since NoV outbreaks can be classified as such with a high degree of confidence based on characteristic epidemiological features (Kaplan et al. 1982).

The datasets from the relevant articles were assigned to three categories depending on the type of data:

- The acronym ‘OutB’ was assigned to articles containing an epidemiological description of a single NoV outbreak;
- Articles providing separate descriptions of diverse NoV outbreaks are labelled as ‘OutB+’;
- Articles with aggregated data, e.g. official register data from entire NoV seasons, were assigned to the ‘AggDat’ category. The main difference to the OutB and OutB+ categories is that articles in the AggDat category do not provide epidemiological data for each single outbreak.

To be able to assess the burden of NoV for personnel on a quantitative basis, we then calculated the proportion of sick employees relative to the total number of NoV cases for each dataset in the four categories by dividing the number of staff with clinical symptoms by the total number of NoV cases. Hence, we obtained a ratio between 0 and 1. A ratio of exactly 1 would, therefore, imply that exclusively employees were involved in a NoV outbreak and vice versa. The proportion of sick staff during the outbreaks was determined with Microsoft Excel™ and the R Software (version 3.5.2).

## Results

In total, 116 datasets were retrieved from 72 relevant articles, whereby 33.6% (*n* = 39) were referring to NoV outbreaks or aggregated data from Germany (Online Resources 1–4). In all, 144,852 persons acquired NoV gastroenteritis in 6,493 outbreaks. According to the epidemiological descriptions in the included articles, 25,408 of them (17.5%) were staff members. A large proportion of 94.0% (*n* = 23,874) were affected by NoV gastroenteritis in hospitals and related settings. Only 1,434 (5.6%) employees fell sick during outbreaks in community facilities. A very minor proportion of them became symptomatic during outbreaks in hotels, restaurants and canteens (*n* = 94; 0.4%) as well as in other food establishments (*n* = 6; 0.0%).

Concerning the proportion of sick staff in each group of outbreaks, we observed different patterns depending on the location of the outbreak. Indeed, the proportion of employees was highest in community facilities. 34.9% (*n* = 1434) of the 4,114 persons who acquired a NoV infection during outbreaks in these settings were employees. Staff members accounted for roughly a fifth (21.4%) of the 111,799 persons suffering from NoV gastroenteritis during outbreaks in health care settings. In contrast, employees account only for 4.6% (*n* = 94) of the 2,052 persons with clinical symptoms during hotel, restaurant and canteen outbreaks. The proportion of sick staff relative to the total number of sick subjects (*n* = 1479) was even lower (0.4%) during outbreaks in other food establishments.

**Fig. 2:**
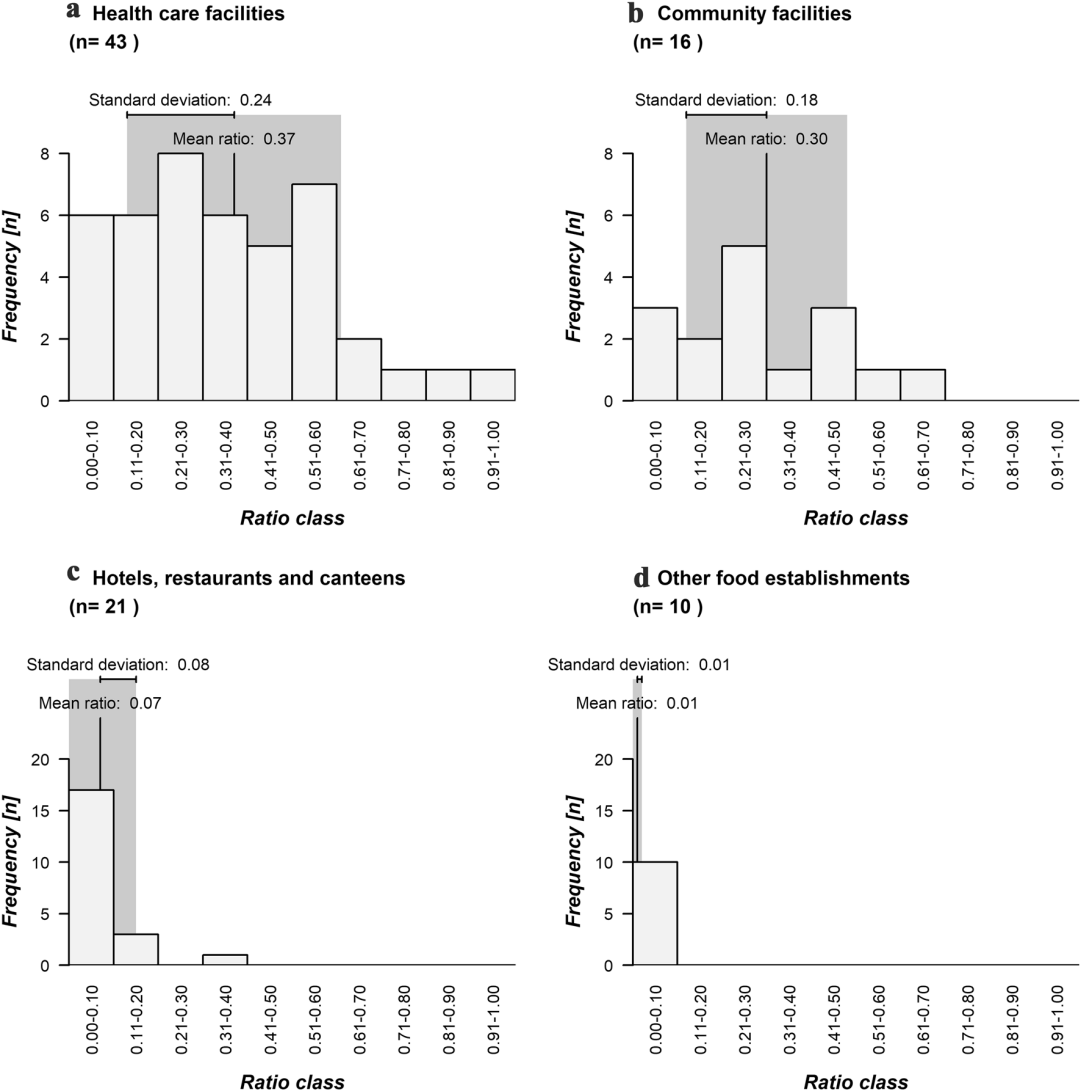
Histograms of ratios between symptomatic staff and total number of persons with clinical symptoms in NoV outbreaks in different settings. The Fig. was created in RStudio 1.1.463

As mentioned above, the ratio between the number of staff members and the total number of persons with clinical symptoms was determined for each dataset, i.e. for both data from single NoV outbreaks and from register data. However, it should be noted that ratios determined for aggregated data should not be compared with that derived for single NoV outbreaks. Indeed, a ratio derived for aggregated data simply reflects the total proportion of sick staff in the whole dataset. However, this number does not provide any information about the variation of the proportion of sick staff during every single outbreak in the dataset. Therefore, it may be possible that an average ratio for whole dataset masks a strong variation in the proportion of sick staff. Due to the limited meaningfulness of the ratios determined for aggregated data, we will only comment on the ratios determined for single NoV outbreaks (*n* = 90, Fig. 2).

Regarding the outbreaks in health care facilities, the number of sick staff and the total number of symptomatic persons was available for 43 NoV outbreaks (Fig. 2). No staff members were affected during five of these epidemics (11.6%). Therefore, a ratio of zero was determined (Online Resource 1). During the majority of the NoV outbreaks in health care facilities (*n* = 25; 58.1%), up to 50% of the symptomatic persons were employees (Fig. 1). A ratio equal or higher than 0.5 was derived for 13 outbreaks (30.2%) (Cunney et al. 2000; Khanna et al. 2003; Baum von et al. 2004; Gallimore et al. 2004; Meyer et al. 2004; Fretz et al. 2005; Zingg et al. 2005; Sukhrie et al. 2012; Schulz-Stübner et al. 2016;). A ratio of exactly one was obtained for a hospital outbreak in Scotland that affected exclusively staff (Vardy et al. 2007). Overall, an average ratio of 0.37 (standard deviation: 0.24) was determined for the outbreaks in the first category (median: 0.35; Fig. 2).

The ratio between the number of symptomatic employees and the total number of NoV cases was determined for 16 NoV outbreaks in community facilities (Fig. 2). Only one published outbreak of NoV gastroenteritis (6.3%) did not affect any staff members (Jian et al. 2012). For three quarters of the epidemics in this category (*n* = 12), a ratio up to 0.5 was obtained. Conversely, staff members were the predominant group among the NoV cases during three remaining outbreaks (18.8%) (Dreier et al. 2006; Sukhrie et al. 2012; Teunis et al. 2015). The highest ratio (0.61) was derived for an outbreak of NoV gastroenteritis in a nursing home (Sukhrie et al. 2012). We observe thus a lower proportion of staff among persons who fell sick due to NoV outbreaks in community facilities. This is also reflected by a lower mean ratio (0.30; standard deviation: 0.18) and median (0.28; Fig. 2).

The relevant publications contained epidemiological descriptions of 21 outbreaks in hotels, restaurants and canteens for which the number of sick employees and the total number of clinical symptoms were reported (Fig. 2). Five of the published outbreaks (23.8%) did not affect any employees (Online Resource 3; Boraja 2008; Guzman-Herrador et al. 2011; O'Neill et al. 2001; Showell et al. 2007; Vo et al. 2016). The proportion of sick staff relative to the total number of NoV cases did not exceed 50% during the remaining outbreaks (*n* = 17; 76.2%). The proportion of sick staff among all persons with clinical symptoms was highest (33%) during a published outbreak in a restaurant (Boxman et al. 2009). A significantly lower mean ratio (0.07; standard deviation: 0.08) and median (0.04) was derived for the epidemics in the third category.

Furthermore, information about the total number of sick persons and sick employees was available for 10 outbreaks in other food establishments (Fig. 2). During the majority of the epidemics (*n* = 6), no staff member fell sick (Online Resource 4; Fretz et al. 2005; Tödt et al. 2006; Zühl 2006), thereby yielding a ratio of zero. Conversely, four outbreaks led to cases of NoV gastroenteritis among employees. The highest ratio (0.04) was obtained for an outbreak in a military base (Wadl et al. 2010). In total, we obtained the lowest mean ratio (0.01; standard deviation 0.01) for this group of outbreaks.

## Discussion

To our knowledge, this is the first contribution in which the significance of NoV in the field of occupational health is highlighted by a systematic review of previously published NoV outbreaks and aggregated data from multiple NoV outbreaks. The review highlights a different proportion of sick staff relative to the total number of affected persons. We hypothesise that these differences are due to different management strategies during NoV outbreaks and a subjectively different experience of the symptoms of a NoV gastroenteritis. This leads to the situation that infectious staff stays away from work for varying lengths of time. Presenteeism may considerably contribute to negative outcomes of NoV outbreaks. An evaluation of the effect of different management strategies on the outcome of NoV outbreaks is beyond the scope of the present study. See, for example, the case study of Vivancos et al. (2010) summarised below for an analysis of the impact of different management strategies on the outcome of NoV outbreaks.

To come back to the initial question as to whether NoV should be considered a significant occupational disease, the results of this review of relevant literature show a differentiated picture. A NoV infection among an immunocompetent subject subsides usually after several days without any remaining damage (Pringle et al. 2015) and most employees are able to restart their work after having recovered (Michaelis et al. 2017). Therefore, NoV should not be considered an occupational disease of major importance. This is also reflected in the data of the German statutory accident insurances between 1991 and 2013, in which NoV gastroenteritis was represented with one single case (oral reference to the statistics of infectious diseases, no. 3101, in the ‘Documentation of occupational diseases in Germany’ database available at https://www.gbe-bund.de (last access: 12 November 2019). As only every fifth person suffering from NoV gastroenteritis was an employee, and that NoV outbreaks in hotels, restaurants, canteens and related settings rarely affected staff, suggest a negligible impact of NoV. On the other hand, more than 50% of the NoV cases during 13 out of 43 outbreaks in health care facilities were employees and those staff members accounted for 34.9% of the NoV cases during community facility outbreaks, it is fair to conclude that NoV should be regarded, from a quantitative perspective, as an important occupational disease in these settings. This is also supported by an earlier study of us. Based on register data from Lower Saxony, we showed that the incidence of NoV among HCWs was significantly higher relative to the incidence of NoV in the total population (Hofmann et al. 2018).

As outlined above, it should always be borne in mind that official statistics and also data from publications do not necessarily reflect the true incidence of NoV due to a significant underreporting (Bernard et al. 2014c) and an inconsistent notification of NoV cases (Hauri et al. 2011). Considering that the etiological agent of gastroenteritis often remains unspecified, underreporting-related biases in our dataset cannot be excluded. Therefore, we stress that all numbers in this paper should be considered carefully. It can be anticipated that underreporting is particularly high in food establishments since the owners of such facilities seek to avoid negative publicity associated with NoV outbreaks and subsequent economic losses. Therefore, it is likely that NoV cases among food handlers often do not show up in official statistics relative to NoV infectious customers.

Consequently, it may be possible that the ratios between the number of sick staff and the total number of symptomatic persons are of limited representativeness, especially in food establishments, thereby challenging the methodological approach of this study. Although this criticism is truly appropriate, it is simply impossible to tackle this problem from a methodological point of view. Due to shortcomings associated with the methodology, underreporting factors may only partly resolve this question (Bernard et al. 2014c). As any conclusive solution is available for this problem, the assessment of the true incidence of NoV will remain a challenge for future prospective studies.

Data from previously published NoV outbreaks indicate that the true importance of NoV lies in its significant economic impact in the form of revenue losses due to bed closures, expenses for microbiological testing and revenue losses due to the absence of sick HCWs, whereby the latter factor seems to be of highest relevance. Indeed, a hospital outbreak in Switzerland involving altogether 16 patients and 29 HCWs led to economic losses in the order of 40,000$, whereby revenue losses due to NoV infections among HCWs accounted for more than 90% of this sum (Zingg et al. 2005). Considering the significantly higher proportion of sick staff during outbreaks in health care and community facilities, it is anticipated that revenue losses due to NoV outbreaks are highest in these settings. It should be noted that the presumably higher revenue losses in community and health care facilities also stem from the fact that the number of employees at risk is significantly higher than in food establishments where the number of employees is usually low.

Based on the significant economic impact of epidemics in health care and community facilities (e.g. Zingg et al. 2005) as well as the considerably higher proportion of sick staff relative to outbreaks in other settings, the compliance with existing infection control measures should increase. Since it has been shown that NoV infectious employees may cause pronounced outbreaks of NoV gastroenteritis (Henke-Gendo et al. 2016; Schulz-Stübner et al. 2016) or contribute to a further spread during ongoing outbreaks (Sideroglou et al. 2014), an exclusion of NoV infectious staff members before the final disappearance of the symptoms should be considered part of a successful prevention strategy. In Germany, for example, §31 and 42 of the Protection against Infection Act grants German public health authorities the right to implement an exclusion policy to ban infectious persons from their work. The associated economic losses should always be viewed in relation to considerably higher costs associated with uncontrolled NoV outbreaks (Schneider et al. 2005).

It should be borne in mind that such a measure alone cannot be regarded as a successful prevention strategy considering the ubiquitous pressure on HCWs due to the chronic staff shortage in hospitals (Ebner and Meyer 2007). Hence, the chronic lack of personnel in hospitals may lead to the paradox situation that the implementation of a well-intentioned prevention strategy is hampered by limited personnel resources. Therefore, the simultaneous implementation of measures against the chronic shortage of staff in hospitals should always be deemed necessary.

One example for such a strategy is a dedicated personnel concept that ensures the availability of sufficient replacement personnel for the event of a NoV outbreak. Such a concept may also help to prevent the presence of sick HCWs at their workplace. A recent own survey among occupational health physicians in hospitals indicated that such concepts exist in some hospitals, but that it is not as ubiquitous as it would be desirable for a successful control of NoV outbreaks. According to the results of our questionnaire survey, only 12 of 53 (23%) interviewed persons declared to be aware of such a concept at their workplace. The low number of interviewed persons may raise the question as to whether the results of the survey can be extrapolated. Nevertheless, they highlight that there is still place for improvement (Michaelis et al. 2017).

A comparison between two regions with differing exclusion policies highlights that a longer exclusion of sick staff may have a positive impact on the course of NoV outbreaks. The outcomes of nursing home outbreaks in one English county with a 48-h exclusion policy for sick employees and another county with a 72-h exclusion policy were compared (Vivancos et al. 2010). The results clearly show that the staff-specific attack rate and the overall attack rate were significantly lower in the latter county. Therefore, it can be anticipated that implementation of the 72-h policy not only caused a reduction of the number of sick staff but also led to a positive impact in the form of reduced revenue losses (Vivancos et al. 2010). It is worth mentioning that these findings are only based on one dataset. Hence, the findings of Vivancos et al. (2010) should only extrapolated with caution.

On the other hand, the implementation of such a 72-h policy is also associated with costs, as replacement personnel needs to be hired. Unfortunately, the authors of did not compare the costs due to the implementation 72-h exclusion policy with the decrease in revenue losses owing to lower attack rates among employees to answer the question as to whether an implementation of a longer exclusion should be considered worth from an economic point of view. A study should be undertaken to answer this question.

At the same time, measures against the chronic understaffing in hospitals may increase the compliance of hygiene measures in health care facilities, since it has been suggested that the chronic staff shortage and the associated lack of time should be regarded as one of the main explanations for the low compliance of hand hygiene measures (Allegranzi and Pittet 2009). The usually low compliance of hygiene measures by HCWs (Creedon 2005) has to be improved. Therefore, specific instructions for HCWs about prevention strategies organised by hygiene specialists before the beginning of the main NoV season could help to enhance the compliance (Henke-Gendo et al. 2016).

Considering that NoV infectious persons may shed the virus for a longer period (Eiffert and Nau 2010), it might be beneficial to allow public health authorities to prolong the exclusion from work if laboratory tests indicate a prolonged shedding of NoV. As we know from our own surveys among nurses (Michaelis et al. 2018) and hygiene specialists (Michaelis et al. 2019), HCWs in hospitals not always remain absent from work while being infected with NoV.

Furthermore, we stress the importance of dedicated outbreak management teams composed of specialists of different disciplines, including occupational health physicians. Such infection control teams should not only ensure a good communication between individuals of different disciplines but monitor the implementation of infection control measures. Among the articles eligible for inclusion we found several examples of NoV outbreaks that were managed by interdisciplinary outbreak management teams (Carpentier et al. 2011; Danial et al. 2016; Einöder-Moreno et al. 2016). Such specialised infection control teams should be the gold standard for the successful management of NoV outbreaks.

We found a low proportion of sick employees during outbreaks in hotels, restaurants, canteens and other food establishments. Indeed, we observe a high number of NoV cases among customers and very sporadic cases among food handlers. Two main factors can be invoked as an explanation for this pattern.

First, food, especially green vegetables (Makary et al. 2009; Patel et al. 2009) or other raw products, as strawberries (Bernard et al. 2014a), that have been contaminated by either food providers or infectious kitchen workers are considered as an effective vector for NoV infections considering the remarkably low infectious dose of NoV (Pringle et al. 2015). Therefore, one or a few NoV infectious food handlers may be sufficient to trigger pronounced NoV outbreaks among customers. Several examples for such outbreaks can be found in the literature (Schmid et al. 2007; Zomer et al. 2010; Vo et al. 2016).

Second, the number of customers exceeds, in most cases, largely the number of food handlers (Johansson et al. 2002; Guzman-Herrador et al. 2011; Mayet et al. 2011). Due to this disproportionality, the ratio between the number of sick staff and the total number of NoV infectious persons should always be close to zero, irrespective of the attack rate among staff. Even a pronounced NoV outbreak leading to an attack rate of 100% among a small group of staff would yield a ratio close to zero. This reasoning suggests that (1) the derived ratios for NoV outbreaks in such facilities should not be regarded as meaningful and that (2) they should not be compared with that of hospital outbreaks, where the size of both groups at risk (HCWs and patients) is usually less disproportional. Considering that the attack rate only reveals information about the proportion of sick persons relative to the total number in a certain group and not about the size of the group itself, the attack rate should also be regarded as an insufficient proxy for the assessment of the burden of NoV.

Based on these considerations, only the proportion of sick staff relative to the total number of NoV cases during outbreaks in food establishments should be regarded as a good proxy for evaluating the burden of NoV outbreaks for staff. As only about 5% of the NoV cases in this group of outbreaks pertain to the personnel, it is deduced that outbreaks of NoV gastroenteritis do not represent a serious problem for the staff themselves, but for persons who are exposed to NoV-contaminated food. Therefore, it should be borne in mind for the design of suitable prevention strategies that they should mainly aim at preventing the spread of NoV from infectious food handlers. Accordingly, an increasing compliance of exclusion policies for NoV-infectious kitchen workers would thus be desirable.

An example from the literature illustrates that an increasing compliance of existing exclusion policies, as §42 of the Protection against Infection act in Germany (RKI 2019a), may lead to a significant reduction of the burden associated with NoV infections. Indeed, a NoV outbreak affecting altogether 52 customers of a canteen would probably have been prevented if the infectious kitchen worker, who was the index case of a NoV outbreak (Schimmelpfennig et al. 2009), would have complied with §42 of the Protection Against Infection act. Therefore, additional incentives are required to increase the compliance of existing regulations. It could be helpful, for example, to offer foodstuff-processing companies compensation fees for the revenue losses associated with the compliance of exclusion policies. Infectious persons could also be assigned to duties other than food handling during a NoV infection, as suggested earlier (Patel et al. 2009), although the complete exclusion of NoV infectious staff remains the most effective strategy for the prevention of NoV outbreaks (Duret et al. 2017).

Our review highlights that there is no consensus about the definition of symptoms of a NoV infection. As mentioned in the methods section, we cannot exclude a bias due to the heterogeneous case definitions in the original publications. Therefore, it would be helpful for future reviews dealing with NoV to define a commonly consented list with clinical symptoms that are indicative for a NoV infection. Furthermore, we observed that the attack rate was rarely reported in the original publications. As this key epidemiological Fig. takes the population at risk into account, the publication of an attack rate in each epidemiological study dealing with NoV would have enabled a more precise assessment of the burden of NoV outbreaks.

Although a NoV vaccine is not yet available owing to several challenges, such as a lack of knowledge about the mechanisms of immunity or the diversity of different NoV strains (Lucero et al. 2018), it would be most relevant for employees suffering themselves from NoV outbreaks or for personnel contributing to a significant spread within the population. According to the vaccination recommendations of the German Permanent Vaccination Commission (STIKO) at the RKI (RKI 2019b), we propose to discuss the relevance of a possible NoV vaccine for the following professional groups:

- employees in health care institutions, including kitchen, laboratory, technical, ambulance and cleaning staff;
- personnel in community facilities (e.g., residential homes, children's day-care centers, children's homes, schools, workshops for disabled persons, asylum-seekers' homes) including kitchen and cleaning staff;
- food handlers in restaurants, canteens or hotels to prevent NoV cases among their customers. This is particularly true for institutions selling food that has previously not been heated.

## Conclusion

The evaluation of previously published NoV outbreaks from the perspective of occupational health enables the following conclusions to be drawn:

- NoV outbreaks lead to significant economic losses due to revenue losses and microbiological testing. Thus, the reduction of the considerable economic impact of NoV should be regarded as the main motivation to increase the compliance with existing hygiene precautions. At the same time, the ubiquitous economic pressure on employees needs to be reduced to ensure a compliance with existing exclusion policies.
- Staff in health care and community facilities seems to be most vulnerable towards NoV outbreaks, whereas NoV cases among food handlers are relatively rare. This pattern is mainly attributed to a comparably higher number of employees in health care and community settings. NoV should thus be regarded as important occupational disease for staff in the former group of settings.
- Regarding the prevention of NoV outbreaks, we strongly advocate the implementation of additional precautions, such as dedicated personnel concepts for NoV outbreaks, to increase the compliance with existing prevention strategies as exclusion policies. The resulting costs should always be viewed relative to significantly higher expenses associated with uncontrolled NoV outbreaks.
- A NoV vaccine would be most beneficial for HCWs that are continuously exposed to NoV infectious persons and for food handlers that may contribute to a significant spread of NoV among larger groups of persons.
- Underreporting-related biases can be regarded as the main limitations of this study and prevent a more precise assessment of the true burden of NoV. We argue that the determination of underreporting factors may only partly resolve this issue. Hence, the estimation of underreporting of NoV cases remains a challenge.

## Electronic supplementary material

Below is the link to the electronic supplementary material.

Supplementary file1 (PDF 495 kb)
