# Supplementary material for: Significance of norovirus in occupational health: a review of published norovirus outbreaks in Central and Northern Europe
Source: Int Arch Occup Environ Health. 2020 May 1;93(8):911–23. doi: 10.1007/s00420-020-01543-4 (PMC7222890; doi:10.1007/s00420-020-01543-4)
Supplement: Supplementary file 1 — Supplementary file1 (PDF 495 kb) [file 420_2020_1543_MOESM1_ESM.pdf]

Supplementary material to

Felix Martin Hofmann [1,2], Edward Olawumi [1], Martina Michaelis[1], Ulrich Stößel [1], Friedrich Hofmann † [1]

Significance of norovirus in occupational health: A review of published norovirus outbreaks in Central and Northern Europe

International Archives of Occupational and Environmental Health

[1] Research Centre for Occupational and Social Medicine (FFAS), Freiburg, Germany

[2] Institute of Earth and Environmental Sciences, University of Freiburg, Germany

Corresponding author

Felix Martin Hofmann

Research Centre for Occupational and Social Medicine (FFAS)

Bertoldstraße 63

D-79098 Freiburg, Germany

Phone +49 761 82526

E-mail: mahofmann@ffas.de

ORCID: 0000-0002-9882-2613

Systematic search for articles on norovirus outbreaks in Central and Northern Europe

EMBASE

Search performed on the 14th of November 2016

1. “Noro*” (English keyword): 51632 articles
2. “Norwalk*” (English keyword): 5553 articles
3. 1 OR 2: 51632 articles
4. “Outbreak*” (English keyword): 2170 articles
5. “Epidemic*” (English keyword): 278838 articles
6. “Pandemic*” (English keyword): 42420 articles
7. “Disease Outbreak*” (English keyword): 86151 articles
8. 4 OR 5 OR 6 OR 7: 291968 articles
9. 3 AND 8: 2651 articles
10. “Personnel” (text word): 367078 articles
11. “Occupational” (text word): 422557 articles
12. “Staff” (text word): 228221 articles
13. „Employee*“ (text word): 47359 articles
14. „Worker*“ (text word): 200973 articles
15. „Health personnel“ (text word): 71345 articles
16. 10 OR 11 OR 12 OR 13 OR 14 OR 15: 1010504 articles
17. 9 AND 16: 320 articles
18. 17 AND AND PY=2000 to 2016 AND LA=(ENGLISH; GERMAN; FRENCH): 284 articles
19. 19 NOT SU=MEDLINE (exclude articles which are listed in MEDLINE): 220 articles

Global Health

Search performed on the 15th of November 2016

1. “Noro*” (English keyword): 2996 articles
2. “Norwalk*” (English keyword): 3365 articles

3. 1 OR 2: 3384 articles

4. “Outbreak*” (English keyword): 26705 articles

5. “Epidemic*” (English keyword): 19030 articles

6. “Pandemic*” (English keyword): 1796 articles

7. “Disease Outbreak*” (English keyword): 15 articles

8. 4 OR 5 OR 6 OR 7: 42649 articles

9. 3 AND 8: 1374 articles

10. “Personnel” (text word): 22661 articles

11. “Occupational” (text word): 52094 articles

12. “Staff” (text word): 25958 articles

13. „Employee*“ (text word): 11893 articles

14. „Worker*“ (text word): 69129 articles

15. „Health personnel“ (text word): 1263 articles

16. 10 OR 11 OR 12 OR 13 OR 14 OR 15: 124171 articles

17. 9 AND 16: 222 articles

18. 17 AND AND PY=2000 to 2016 AND LA=(ENGLISH; GERMAN; FRENCH): 190 articles

GMS

Search performed on the 13th of November 2016

1. “Noro*” (text word): 13 articles

2. “Norwalk” (text word): 2 articles

3. 1 OR 2: 14 articles

4. “Outbreak*” (text word): 50 articles

5. “Epidemic*” (text word): 35 articles

6. “Pandemic*” (text word): 6 articles

7. “Disease Outbreak*” (text word): 2 articles

8. 4 OR 5 OR 6 OR 7: 75 articles

9. 8 AND LA=(ENGLISH; GERMAN; FRENCH): 5 articles

10. 9 AND PY=2000 to 2016: 5 articles

GMS meetings

Search performed on the 13th of November 2016

1. “Noro*” (text word): 10 articles
2. “Norwalk” (text word): 2 articles
3. 1 OR 2: 11 articles
4. “Outbreak*” (text word): 43 articles
5. “Epidemic*” (text word): 53 articles
6. “Pandemic*” (text word): 10 articles
7. “Disease Outbreak*” (text word): 0 articles
8. 4 OR 5 OR 6 OR 7: 93 articles
9. 3 AND 8: 1 article

PUBMED

Search carried out on the 17th of October 2016

1. “Noro*” (MeSH terms): 3549 articles
2. “Norwalk virus” (MeSH terms): 679 articles
3. 1 OR 2: 3549 articles
4. “Outbreak*” (text word): 104085 articles
5. “Epidemic*” (text word): 84811 articles
6. “Pandemic* (text word): 20435 articles
7. “Disease outbreak*” (text word): 71231 articles
8. 4 AND 5 AND 6 AND 7: 179673 articles
9. 3 AND 8: 1717 articles
10. “french” (language) OR “german” (language) OR “english” (language): 23498916 articles
11. 9 AND 10: 1577 articles

12. 11 AND “2000/01/01” (date - publication): “3000” (date - publication): 1366 articles
13. “personnel” (text word): 298546 articles
14. “occupational” (text word): 244747 articles
15. “staff” (text word): 197535 articles
16. “employee*” (text word): 53030 articles
17. “worker*” (text word): 157446 articles
18. “Health personnel” (MeSH term): 424846 articles
19. 13 OR 14 OR 15 OR 16 OR 17 OR 18: 988212 articles
20. 12 AND 19: 186 articles

Outbreak Database

Search performed on the 11th of October 2016

GE=:"Norovirus" (GE: microorganism, genus) AND RY:[2000 TO 2147483647] (RY: publication year) AND NOC=:"Personnel" (group that suffered from the outbreak) AND (FY=:"Inpatient care (not ICU)" OR FY=:"Intensive care unit" OR FY=:"Nursing home") (FY: facility): 37 results

Online Resource 1: Epidemiological data for NoV outbreaks in health care facilities

Authors/year of publ.	Study type[1]	Country[2]	Setting	Transmission path[3]	Genogroup (GG)	Genotype	Collectives infected	Outbreak year(s)	Outbreak(s) [n]	Subjects with symptoms [n]	Employees affected [n]	Subjects (non-staff with symptoms) [n]	Ratio symptomatic employees: symptomatic persons (total)	Remarks
Cummins and Ready (2016)	AggDat	GB	Hospitals (n=4)	(---)[4]	GGI & GGII	(---)	Patients & HCWs	2015	2	4	0	4	0.00	
					GGII				1	3	0	3	0.00	
					GGII				16	51	7	44	0.14	
					GGII				1	6	0	6	0.00	
Danial et al. (2016)	OutB	GB	Hospital	(---)	GGII	GGII.4 Sydney 2012	Patients & HCWs	2013	1	173	30	143	0.17	
Harris (2016)	AggDat	GB	Hospitals (n=?)	(---)	(---)	(---)	Patients & HCWs	2009 - 2016	5160	91814	18685	73129	0.20	
Schulz-Stübner et al. (2016)	OutB+	DE	Hospitals (n=?)	EC, p2p	(---)	(---)	Patients & HCWs	2013-2015	1	11	6	5	0.55	
									1	100	34	66	0.34	
									1	10	3	7	0.30	
									1	7	4	3	0.57	
									1	13	2	11	0.15	
									1	10	6	4	0.60	
									1	10	4	6	0.40	
									1	11	2	9	0.18	
									1	12	3	9	0.25	

Authors/year of publ.	Study type[1]	Country[2]	Setting	Transmission path[3]	Genogroup (GG)	Genotype	Collectives infected	Outbreak year(s)	Outbreak(s) [n]	Subjects with symptoms [n]	Employees affected [n]	Subjects (non-staff with symptoms) [n]	Ratio symptomatic employees: symptomatic persons (total)	Remarks
Mattner et al. (2015)	AggDat	DE	Hospitals (n=5)	EC, p2p	(---)	(---)	Patients & HCWs	2002/ 2003	5	130	46	84	0.35	
								2002/ 2003	13	382	83	299	0.22	
								2007- 2009	24	442	129	313	0.29	
								2010- 2012	11	181	36	145	0.20	
								2010- 2012	18	297	54	243	0.18	
Mitchell et al. (2015)	AggDat	GB	Hospitals (n=5)	(---)	(---)	(---)	Patients & HCWs	2007/ 2008	59	694	87	607	0.13	
								2008/ 2009	31	324	40	282	0.12	
								2009/ 2010	21	272	54	218	0.20	
								2010/ 2011	3	43	20	23	0.47	
								2011/ 2012	2	32	11	21	0.34	
								2012/ 2013	2	25	8	17	0.32	
								2013/ 2014	1	10	1	9	0.10	

Authors/year of publ.	Study type[1]	Country[2]	Setting	Transmission path[3]	Genogroup (GG)	Genotype	Collectives infected	Outbreak year(s)	Outbreak(s) [n]	Subjects with symptoms [n]	Employees affected [n]	Subjects (non-staff with symptoms) [n]	Ratio symptomatic employees: symptomatic persons (total)	Remarks
Rahamat-Langendoen et al. (2013)-	OutB	NL	Hospital	(---)	GGII	GGII.4 2009, GGII.b, GGII.7, GGII.2	Patients & HCWs	(---)	1	9	2	7	0.22	
Sauvan et al. (2013)	OutB	CH	Hospital	(---)	(---)	(---)	Patients & HCWs	2012-2013	1	163	33	130	0.20	
Heijne et al. (2012)	OutB	NL	Psychiatric institution	p2p	(---)	(---)	Patients & HCWs	2008	1	46	13	33	0.28	
Sukhrie et al. (2012)	OutB+	NL	Hospitals (n=3)	(---)	GGII	GGII.4-2008/GGII.4.-2006b	Patients & HCWs	2009	1	16	11	5	0.69	
						GGII.2		2009	1	6	0	6	0.00	
						GGII.7		2009	1	8	7	1	0.88	
Carpentier et al. (2011)	OutB	FR	Hospital	(---)	GGII	GGII.4	Patients & HCWs	2008	1	150	43	107	0.29	
Danial et al. (2011)	AggDat	GB	Hospitals (n=?)	(---)	(---)	(---)	Patients & HCWs	2007-2009	142	2317	599	1718	0.26	
Hauri et al. (2011)	AggDat	DE	Hospitals (n=?)/rehabilitation centers (n=?)	(---)	(---)	(---)	Patients & HCWs	2007	155	3381	660	2721	0.20	
Illingworth et al. (2011)	AggDat	GB	Hospital and community	(---)	(---)	(---)	Patients & HCWs	2009/ 2010	24	318	60	258	0.19	[1]

Authors/year of publ.	Study type[1]	Country[2]	Setting	Transmission path[3]	Genogroup (GG)	Genotype	Collectives infected	Outbreak year(s)	Outbreak(s) [n]	Subjects with symptoms [n]	Employees affected [n]	Subjects (non-staff with symptoms) [n]	Ratio symptomatic employees: symptomatic persons (total)	Remarks
Schmid et al. (2011)	OutB	AT	Hospital with affiliated rehabilitation center and convalescent home	Food, p2p, EC	GGII	GGII.4 2006b	Patients & HCWs	2009	1	204	52	152	0.25	
Schwartz et al. (2011)	OutB	DE	Hospital	p2p	GGII	GGII.4	Patients & HCWs	(---)	1	37	11	26	0.30	
Aziz (2010)	AggDat	GB	Hospitals (n=?)	(---)	(---)	(---)	Patients & HCWs	2009	458	5746	1265	4481	0.22	
Fretz et al. (2009)	OutB	AT	Hospital	p2p, EC	GGII	GGII.4 2006b	Patients & HCWs	2006/ 2007	1	70	14	56	0.20	
Kanerva et al. (2009)	OutB	FI	Hospital	p2p	GGII	GGII.4 2006b	Patients & HCWs	2006	1	445	205	240	0.46	
Sommer et al. (2009)	OutB+	AT	Hospitals (n=2)	(---)	(---)	(---)	Patients (infants) & HCW	2007/ 2008	1	2	1	1	0.50	
					GGII				1	5	0	5	0.00	
Leuenberger et al. (2007)	OutB	CH	Hospital	p2p, EC	GGII	GGII.4	Patients & HCWs	2003	1	77	28	49	0.36	
Vardy et al. (2007)	OutB	GB	Hospital	(---)	(---)	(---)	HCWs	2006	1	37	37	0	1.00	
Mattner et al. (2006)	OutB	DE	Hospital	p2p	GGII	GGII.4 Grimsby	Patients & HCWs	2002	1	163	79	84	0.48	

Authors/year of publ.	Study type[1]	Country[2]	Setting	Transmission path[3]	Genogroup (GG)	Genotype	Collectives infected	Outbreak year(s)	Outbreak(s) [n]	Subjects with symptoms [n]	Employees affected [n]	Subjects (non-staff with symptoms) [n]	Ratio symptomatic employees: symptomatic persons (total)	Remarks
Simon et al. (2006)	OutB	DE	Hospital	(---)	(---)	(---)	Patients and relatives thereof	2004	1	13	0	13	0.00	
Fretz et al. (2005)	OutB+	CH	Hospital and affiliated nursing home	p2p, food	GGII	GGII.4 Miami Beach	Patients & HCWs	2003	1	140	78	62	0.56	
Odelin et al. (2005)	OutB	FR	Hospital	p2p	GGII	GGII.4 Lordsdale	Patients & HCWs	2002	1	110	40	70	0.36	
Schmid et al. (2005)	OutB+	AT	Hospital and nursing home	p2p, EC	GGII	GGII.4 (Jamboree like)	Patients & HCW s	2004	1	53	25	28	0.47	
Zingg et al. (2005)	OutB	CH	Hospital	(---)	(---)	(---)	Patients & HCWs	2003	1	45	29	16	0.64	
Baum von et al. (2004)	OutB	DE	Hospital	(---)	(---)	(---)	Patients & HCWs	2004	1	16	12	4	0.75	
Gallimore et al. (2004)	OutB	GB	Hospital	(---)	GGI, GGII	GGI.h Potsdam, GGII.3a Harrow/Mexico, GGII.7 Leeds, GGII.4 Grimsby	Patients & HCWs	2002	1	99	59	40	0.60	
Lopman et al. (2004)	AggDat	GB	Hospitals (n=15)	(---)	(---)	(---)	Patients & HCWs	2002/ 2003	61	1212	482	730	0.40	
Jansen et al. (2004)	OutB	DE	Hospital and nursing home	p2p , EC, aer	GGII	GGII.4 Grimsby-like	Patients & HCWs	2002/ 2003	1	322	137	185	0.43	[2]
Meyer et al. (2004)	OutB	DE	Hospital	(---)	(---)	(---)	Patients & HCWs	2002	2	95	67	28	0.71	

Authors/year of publ.	Study type[1]	Country[2]	Setting	Transmission path[3]	Genogroup (GG)	Genotype	Collectives infected	Outbreak year(s)	Outbreak(s) [n]	Subjects with symptoms [n]	Employees affected [n]	Subjects (non-staff with symptoms) [n]	Ratio symptomatic employees: symptomatic persons (total)	Remarks
Verbelen et al. (2004)	OutB	BE	Hospital	(---)	GGII	GGII.4 Camberwell	Patients & HCWs	2002	1	31	11	20	0.35	
Khanna et al. (2003)	OutB	CH	Hospital	p2p	GGII	GGII._ Basel	Patients & HCW s	2001	1	63	36	27	0.57	
Billgren et al. (2002)	AggDat	SE	Hospitals (n=10)	aer, p2p, EC	GGI, GGII	GGI.2 Hawaii, GGII.3 Mexico, GGII.4 Lordsdale	Patients & HCWs	1996	54	794	237	557	0.30	
Kuusi et al. (2002)	OutB	FI	Rehabilitation center	p2p	GGII	(---)	Patients, HCWs, administrative staff, kitchen, cleaning staff & maintenance staff	1999/ 2000	1	331	118	213	0.36	
O'Neill et al. (2001)	OutB+	GB	Hospital	(---)	(---)	(---)	Patients & HCWs	1999	1	30	9	21	0.30	
Oppermann et al. (2001)	OutB	DE	Hospital	p2p	(---)	(---)	Patients & HCWs	1999	1	83	11	72	0.13	
Cunney et al. (2000)	OutB	IE	Hospital	p2p,food,aer	(---)	(---)	Patients, HCWs & food handlers	1993	1	95	48	47	0.51	

Legend

[1]Study type: Aggregated data of diverse NoV outbreaks (aggDat), outbreak case study (OutB), outbreak case studies, i.e. more than one NoV outbreak that are separately reported in a publication (OutB+)

[2]Country according to ISO 3166-1: Austria (AT), Belgium (BE), Germany (DE), Finland (FI), France (FR), United Kingdom (GB), Ireland (IE), the Netherlands (NL), Norway (NO), Sweden (SE)

[3]Transmission path: aerosol (aer), environmental contamination (EC), person-to-person (p2p)

[4]Not specified

[1] Fragmentary data for the 2007/2008 season, therefore excluded; [2] only data available for one nursing home/ hospital outbreak

Online Resource 2: Epidemiological data for NoV outbreaks in community facilities

Authors/year of publ.	Study type[1]	Country[2]	Setting	Transmission path[3]	Genogroup	Genotype	Collectives infected	Outbreak year(s)	Outbreak(s) [n]	Subjects with symptoms [n]	Employees affected [n]	Subjects (non-staff with symptoms) [n]	Ratio symptomatic employees: symptomatic persons (total)	Remarks
Loury et al. (2015)	OutB	FR	Nursing home	Food	GGII	GGII.g/GGII.1, GGII.7	Residents & HCWs	2012	1	84	31	53	0.37	
Teunis et al. (2015)	OutB+	NL	Nursing home	?	(---)[4]	(---)	Residents & HCWs	2009-2012	1	19	11	8	0.58	[1]
Jian et al. (2012)	OutB	AT	School	Food, p2p	GGII	GGII.6/GGII.7 Hybrid	Students	2011	1	48	0	48	0.00	
Sukhrie et al. (2012)	OutB+	NL	Nursing home	(---)	GGII	GGII.4 2010	Residents & HCWs	2009	1	18	11	7	0.61	[2]
								2009	1	17	8	9	0.47	
Vivancos et al. (2010)	AggDat	GB	Nursing homes & residential homes (n=?)	(---)	GGII, GGI (?)	(---)	Residents & HCWs	2006-2007	96	2114	727	1387	0.34	[3]
Friesema et al. (2009)	AggDat	NL	Nursing homes (n=49)	(---)	GGI, GGII	GGII.4 2004, GGII.4 2006a, GGII2006b, GGI.3 Birmingham, GGI.6 Mikkeli, GGII.b Hilversum, GGII.3 Toronto, GGII.7 Leeds	Residents & HCWs	2005-2007	26	1027	390	637	0.38	
Adams (2008)	OutB	DE	Nursing home	(---)	(---)	(---)	Residents & HCWs	2007	1	50	15	35	0.30	
Kaynak et al. (2007)	OutB	DE	Nursing home	(---)	(---)	(---)	Residents & HCWs	2006	1	84	19	65	0.23	

Authors/year of publ.	Study type[1]	Country[2]	Setting	Transmission path[3]	Genogroup	Genotype	Collectives infected	Outbreak year(s)	Outbreak(s) [n]	Subjects with symptoms [n]	Employees affected [n]	Subjects (non-staff with symptoms) [n]	Ratio symptomatic employees: symptomatic persons (total)	Remarks
Dreier et al. (2006)	OutB+	DE	Nursing home	p2p	GGI	GGI.4 Chiba	Residents & HCWs	2004	1	17	5	12	0.29	
			Nursing home	p2p	GGI	GGI.4 Chiba	Residents & HCWs		1	12	5	7	0.42	
			Residential home	p2p	GGI	GGI.2 Southampton	Patients & HCWs		1	24	6	18	0.25	
			Residential home	p2p	GGI	GGI.2 Southampton	Patients & HCWs		1	22	6	16	0.27	
			Residential home	p2p	GGI	GGI.2 Southampton	Patients & HCWs		1	36	3	33	0.08	
			Residential home	p2p	GGI	GGI.2 Southampton	Patients & HCWs		1	10	2	8	0.20	
			Residential home	p2p	GGI	GGI.2 Southampton	Patients & HCWs		1	40	20	20	0.50	
Lopman et al. (2004)	AggDat	GB	Nursing homes (n=135)	(---)	(---)	(---)	Patients & HCWs	2002/ 2003	14	432	166	266	0.38	
O'Neill et al. (2001)	OutB+	GB	Nursery school	(---)	(---)	(---)	Pupils & school staff	(---)	1	25	2	23	0.08	
			Nursing home				Residents & HCWs		1	35	7	28	0.20	

Legend

1-4 see supplementary table 1 [1] also asymptomatic subjects tested for NoV, four of five outbreaks in the article were already published in Sukhrie et al. (2012); [2] also asymptomatic subjects tested for NoV; [3] only 34 of 96 gastroenteritis outbreaks are confirmed NoV outbreaks

Online Resource 3: Epidemiological data for NoV outbreaks in hotels, restaurants and canteens

Authors/year of publ.	Study type[1]	Country[2]	Setting	Transmission path[3]	Genogroup	Genotype	Collectives infected	Outbreak year(s)	Outbreak(s) [n]	Subjects with symptoms [n]	Employees affected [n]	Subjects (non-staff with symptoms) [n]	Ratio symptomatic employees: symptomatic persons (total)	Remarks
Brey (2016)	OutB	DE	Restaurant	Food?	GGI	GGI.4	Diners & cooks	2016	1	50	2	48	0.04	
Vo et al. (2016)	OutB	FI	Restaurant	Food?	GGI	GGI.7	Diners & relatives of food handlers	2015	1	15	0	15	0.00	[1]
Maritschnik et al. (2013)	OutB	AT	Hotel	Food	GGII	GGII.4 Sydney 2012	Diners & cooks	2012	1	27	1	26	0.04	
Smith et al. (2012)	OutB	GB	Restaurant	Food, p2p, EC?	GGII	GGII.2, GGII.3, GGII.4, GGII.6	Diners, kitchen staff, front of house staff, sommeliers, administrators & kitchen porters	2009	1	257	17	240	0.07	
Baker et al. (2011)	OutB	GB	Restaurant	Food	GGI, GGII	(---)[4]	Diners & kitchen staff	2010	1	12	1	11	0.08	

Authors/year of publ.	Study type[1]	Country[2]	Setting	Transmission path[3]	Genogroup	Genotype	Collectives infected	Outbreak year(s)	Outbreak(s) [n]	Subjects with symptoms [n]	Employees affected [n]	Subjects (non-staff with symptoms) [n]	Ratio symptomatic employees: symptomatic persons (total)	Remarks
Guzman-Herrador et al. (2011)	OutB	NO	Hotel	Food?	(---)	(---)	Meeting participants	2011	1	56	0	56	0.00	
Nicolay et al. (2011)	OutB	IE	Hotel	Food?	GGII	GGII.4 2006	Diners & food handlers	2009	1	28	1	27	0.04	[2]
Boxman et al. (2009)	OutB+	NL	Restaurant	Food	GGI	GGI.6	Diners & food handlers	2007	1	21	7	14	0.33	
Kuo et al. (2009)	OutB	AT	Restaurant	Food	GGII	(---)	Diners & kitchen staff	2007	1	21	2	19	0.10	
Makary et al. (2009)	OutB	FI	Canteen (n=3)	Food	GGII	GGII.1	Employees, cooks	2006	3	114	3	111	0.03	
Schimmelpfennig et al. (2009)	OutB	DE	Restaurant	Food	(---)	(---)	Diners & kitchen staff	2007	1	53	1	52	0.02	
Boraja (2008)	OutB	DE	Restaurant	(---)	(---)	(---)	Diners	2007	1	20	0	20	0.00	
Sinn (2008)	OutB	DE	Restaurant	EC, p2p,food(?)	GGII	GGII.4 2006b	Diners & kitchen staff	2006	1	23	2	21	0.09	
Fell et al. (2007)	OutB	DE	Canteen	Food	GGI	(---)	Diners & kitchen staff	2005	1	70	1	69	0.01	

Authors/year of publ.	Study type[1]	Country[2]	Setting	Transmission path[3]	Genogroup	Genotype	Collectives infected	Outbreak year(s)	Outbreak(s) [n]	Subjects with symptoms [n]	Employees affected [n]	Subjects (non-staff with symptoms) [n]	Ratio symptomatic employees: symptomatic persons (total)	Remarks
Michel et al. (2007)	OutB	IE	Hotel	p2p	(---)	(---)	Diners & cooks	2006	1	98	11	87	0.11	
Showell et al. (2007)	OutB	GB	Canteen	Food	GGII	GGII.4 v2, GGII.4 v3	Diners & food handlers	2007	1	36	0	36	0.00	
Wit de et al. (2007)	OutB	NL	Restaurant	Food	GGII	(---)	Diners & cooks	2001	1	249	18	231	0.07	
Johansson et al. (2002)	OutB	SE	Hotel	Food, p2p, EC	GGI	GGI.3 Desert Shield	Hotel guests & hotel staff	2000	1	201	9	192	0.04	
O'Neill et al. (2001)	OutB+	GB	Hotel	(---)	(---)	(---)	Hotel guests & hotel staff	1998	1	186	14	172	0.08	
			Hotel	(---)	(---)	(---)	Hotel guests & catering staff	1999	1	23	3	20	0.13	
			Hotel	(---)	(---)	(---)	Hotel guests	2000	1	485	0	485	0.00	
			Restaurant	(---)	(---)	(---)	Diners & cooks	1999	1	7	1	6	0.14	

Legend: [1-4] see supplementary table 1;

[1] Only stool samples submitted by kitchen workers (without symptoms?) and relatives thereof;

[2] also asymptomatic persons tested for NoV

Online Resource 4: Epidemiological data for NoV outbreaks in other settings associated with food handling

Authors/year of publ.	Study type[1]	Country[2]	Setting	Transmission path[3]	Genogroup	Genotype	Collectives infected	Outbreak year(s)	Outbreak(s) [n]	Subjects with symptoms [n]	Employees affected [n]	Subjects (non-staff with symptoms) [n]	Ratio symptomatic employees: symptomatic persons (total)	Remarks
Einöder-Moreno et al. (2016)	OutB	NO	Conference centre	Food	GGI	GGI.2 Southhampton	Meeting participants, kitchen staff	2013	1	76	2	74	0.03	
Mayet et al. (2011)	OutB	FR	Military camp	Food	GGI	(---)[4]	Military personnel, cook	2011	1	148	1	147	0.01	
Wadl et al. (2010)	OutB	DE	Military base	Food, p2p, EC	GGII	GGII.4 2006b	Military personnel, kitchen staff	2009	1	28	1	27	0.04	[1]
Zomer et al. (2010)	OutB	SE	Manufacturing company	Food	GGI	GGI.3 Desert Shield	Employees, food handlers	2007	1	308	2	306	0.01	
	OutB+	DE	Company excursion	Food	GGII	GGII.2 Melksham-like	Excursion participants	2006	1	73	0	73	0.00	[2]
			Seminar	Food	GGII	GGII.2 Melksham-like	Seminar participants		1	21	0	21	0.00	
			Social gathering	Food	(---)	(---)	Social gathering participants		1	45	0	45	0.00	
Zühl (2006)	OutB	DE	Broadcasting center	(---)	(---)	(---)	Employees, security staff	2006	1	47	0	47	0.00	
Fretz et al. (2005)	OutB+	CH	Municipality (n=2)	Water	GGI, GGII	GGI.2 White Rose, GGII.4 Camberwell	Inhabitants	2001	1	>650	0	>650	0.00	

Authors/year of publ.	Study type1	Country2	Setting	Transmission path3	Genogroup	Genotype	Collectives infected	Outbreak year(s)	Outbreak(s) [n]	Subjects with symptoms [n]	Employees affected [n]	Subjects (non-staff with symptoms) [n]	Ratio symptomatic employees: symptomatic persons (total)	Remarks
			Ski camp (n=3)	Food, EC	(---)	(---)	Skiers		3	63	0	63	0.00	
			Social gathering	(---)	GGI	GGI.4 Chiba	Family members		1	20	0	20	0.00	

Legend

1-4 see supplementary table 1

[1] the symptomatic staff member reported only nausea; [2] all three NoV outbreaks are linked to the same food handler

References

Adams B (2008) [On multiple norovirus outbreaks in the district of Olpe]. Zu mehreren Norovirus-Ausbrüchen im Kreis Olpe Epidemiologisches Bulletin 6:48–49

Aziz A-M (2010) Managing outbreaks of norovirus in an NHS hospital Brit J Nurs 19:589–596

Baker K, Morris J, McCarthy N, Saldana L, Lowther J, Collinson A, Young M (2011) An outbreak of norovirus infection linked to oyster consumption at a UK restaurant, February 2010 J Public Health (Oxf) 33:205–211

Baum von H, Kochs M, Gonser T (2004) [On an outbreak of norovirus-related diseases in a hospital]. Zu einem Ausbruch Norovirus-bedingter Erkrankungen in einem Klinikum Epidemiologisches Bulletin 36:302

Billgren M, Christenson B, Hedlund KO, Vinjé J (2002) Epidemiology of Norwalk-like Human Caliciviruses in Hospital Outbreaks of Acute Gastroenteritis in the Stockholm Area in 1996 J Infect 44:26–32

Boraja G (2008) [On a norovirus outbreak during a school trip]. Zu einem Norovirus-Ausbruch während einer Klassenfahrt Epidemiologisches Bulletin 6:48

Boxman I, Dijkman R, Verhoef L, Maat A, Dijk van G, Vennema H, Koopmans M (2009) Norovirus on Swabs Taken from Hands Illustrate Route of Transmission: A Case Study J Food Prot 72:1753–1755

Brey R (2016) [Norovirus outbreak in the district of Amberg-Sulzbach, Bavaria, September 2016]. Norovirus-Ausbruch im Landkreis Amberg-Sulzbach, Bayern, September 2016 Epidemiologisches Bulletin 48:531–534

Carpentier M et al. (2011) Investigation and Control of a Nosocomial Norovirus Outbreak in a Long-Term Care Facility Infect Control Hosp Epidemiol 32:1052–1055

Cummins M, Ready D (2016) Role of the Hospital Environment in Norovirus Containment J Infect Dis 213:12–14

Cunney RJ et al. (2000) Investigation of an outbreak of gastroenteritis caused by Norwalk-like virus, using solid phase immune electron microscopy J Hosp Infect 44:113–118

Danial J et al. (2016) Lessons learned from a prolonged and costly norovirus outbreak at a Scottish medicine of the elderly hospital: case study J Hosp Infect 93:127–134

Danial J, Cepeda JA, Cameron F, Cloy K, Wishart D, Templeton KE (2011) Epidemiology and costs associated with norovirus outbreaks in NHS Lothian, Scotland 2007–2009 J Hosp Infect 79:354–358

Dreier J, Störmer M, Mäde D, Burkhardt S, Kleesiek K (2006) Enhanced Reverse Transcription-PCR Assay for Detection of Norovirus Genogroup I J Clin Microbiol 44:2714–2720

Einöder-Moreno M, Lange H, Grepp M, Osborg E, Vainio K, Vold L (2016) Non-heat-treated frozen raspberries the most likely vehicle of a norovirus outbreak in Oslo, Norway, November 2013 Epidemiol Infect 144:2765–2772

Fell G, Boyens M, Baumgarte S (2007) [Frozen berries as a risk factor for outbreaks of norovirus gastroenteritis. Results of an outbreak investigation in the summer of 2005 in Hamburg]. Tiefkühlfrüchte als Risikofaktor für Gastroenteritis-Ausbrüche durch Noroviren Bundesgesundheitsbla 50:230–236

Fretz R et al. (2009) An outbreak of norovirus gastroenteritis in an Austrian hospital, winter 2006–2007 Wien Klin Wochenschr 121:137–143

Fretz R, Svoboda P, Lüthi TM, Tanner M, Baumgartner A (2005) Outbreaks of gastroenteritis due to infections with Norovirus in Switzerland, 2001–2003 Epidemiol Infect 133:429–437

Friesema IHM et al. (2009) Differences in clinical presentation between norovirus genotypes in nursing homes J Clin Virol 46:341–344

Gallimore CI, Cubitt D, du Plessis N, Gray JJ (2004) Asymptomatic and Symptomatic Excretion of Noroviruses during a Hospital Outbreak of Gastroenteritis J Clin Microbiol 42:2271–2274

Guzman-Herrador B, Heier BT, Osborg EJ, Nguyen VH, Vold L (2011) Outbreak of norovirus infection in a hotel in Oslo, Norway, January 2011 Euro Surveill 16:19928

Harris JP (2016) Norovirus Surveillance: An Epidemiological Perspective J Infect Dis 213:S8–S11

Hauri AM, Westbrock H-J, Claus H, Geis S, Giernat S, Forβbohm M, Uphoff H (2011) Electronic Outbreak Surveillance in Germany: A First Evaluation for Nosocomial Norovirus Outbreaks PLOS ONE 6:e17341

Heijne JCM et al. (2012) Quantifying Transmission of Norovirus During an Outbreak Epidemiology 23:277–284

Illingworth E, Taborn E, Fielding D, Cheesbrough J, Diggle PJ, Orr D (2011) Is closure of entire wards necessary to control norovirus outbreaks in hospital? Comparing the effectiveness of two infection control strategies J Hosp Infect 79:32–37

Jansen A et al. (2004) [Outbreak of Norovirus in Berlin - Epidemiological and Clinical Features and Prevention]. Die Norovirus-Epidemie in Berlin - Klinik, Epidemiologie und Prävention Z Gastroenterol 42:311–316

Jian S-W, Simons E, Lederer I, Hoehne M, Offner E, Allerberger F, Schmid D (2012) A foodborne outbreak due to norovirus in a vocational school, Austria November 2011 Wien Tierarztl Monatsschr 99:24–29

Johansson PJH, Torvén M, Hammarlund A-C, Björne U, Hedlund K-O, Svensson L (2002) Food-Borne Outbreak of Gastroenteritis Associated with Genogroup I Calicivirus J Clin Microbiol 40:794–798

Kanerva M, Maunula L, Lappalainen M, Mannonen L, von Bonsdorff CH, Anttila VJ (2009) Prolonged norovirus outbreak in a Finnish tertiary care hospital caused by GII.4-2006b subvariants J Hosp Infect 71:206–213

Kaynak S, Pohl S, Kubasch S (2007) [On a norovirus outbreak in a nursing home]. Zu einem Norovirus-Ausbruch in einem Pflegeheim Epidemiologisches Bulletin 9:71–72

Khanna N, Goldenberger D, Graber P, Battegay M, Widmer AF (2003) Gastroenteritis outbreak with norovirus in a Swiss university hospital with a newly identified virus strain J Hosp Infect 55:131–136

Kuo H-W, Schmid D, Jelovcan S, Pichler A-M, Magnet E, Reichart S, Allerberger F (2009) A Foodborne Outbreak Due to Norovirus in Austria, 2007 J Food Prot 72:193–196

Kuusi M, Nuorti JP, Maunula L, Tran Minh NN, Ratia M, Karlsson J, Von Bonsdorff CH (2002) A prolonged outbreak of Norwalk-like calicivirus (NLV) gastroenteritis in a rehabilitation centre due to environmental contamination Epidemiol Infect 129:133–138

Leuenberger S, Widdowson M-A, Feilchenfeldt J, Egger R, Streuli RA (2007) Norovirus outbreak in a district general hospital - new strain identified Swiss Med Wkly 137:57–61

Lopman BA et al. (2004) Epidemiology and Cost of Nosocomial Gastroenteritis, Avon, England, 2002–2003 Emerging Infect Dis 10:1827–1834

Loury P, Le Guyader FS, Le Saux JC, Ambert-Balay K, Parrot P, Hubert B (2015) A norovirus oyster-related outbreak in a nursing home in France, January 2012 Epidemiol Infect 143:2486–2493

Makary P et al. (2009) Multiple norovirus outbreaks among workplace canteen users in Finland, July 2006 Epidemiol Infect 137:402–407

Maritschnik S et al. (2013) A Food Handler-Associated, Foodborne Norovirus GII.4 Sydney 2012-Outbreak Following a Wedding Dinner, Austria, October 2012 Food Environ Virol 5:220–225

Mattner F, Guyot A, Henke-Gendo C (2015) Analysis of norovirus outbreaks reveals the need for timely and extended microbiological testing J Hosp Infect 91:332–337

Mattner F, Sohr D, Heim A, Gastmeier P, Vennema H, Koopmans M (2006) Risk groups for clinical complications of norovirus infections: an outbreak investigation Clin Microbiol Infect 12:69–74

Mayet A et al. (2011) Food-borne outbreak of norovirus infection in a French military parachuting unit, April 2011 Euro Surveill 16:19930

Meyer E, Ebner W, Scholz R, Dettenkofer M, Daschner FD (2004) Nosocomial outbreak of norovirus gastroenteritis and investigation of ABO histo-blood group type in infected staff and patients J Hosp Infect 56:64–66

Michel A, Fitzgerald R, Whyte D, Fitzgerald A, Beggan E, O'Connell N, Greally T (2007) Norovirus outbreak associated with a hotel in the west of Ireland, 2006 Euro Surveill 12:19931

Mitchell C, Meredith P, Richardson M, Greengross P, Smith GB (2015) Reducing the number and impact of outbreaks of nosocomial viral gastroenteritis: time-series analysis of a multidimensional quality improvement initiative BMJ Qual Saf 6:466–474

Nicolay N et al. (2011) Potential role of asymptomatic kitchen food handlers during a food-borne outbreak of norovirus infection, Dublin, Ireland, March 2009 Euro Surveill 16:19931

O'Neill HJ, McCaughey C, Wyatt DE, Mitchell F, Coyle PV (2001) Gastroenteritis outbreaks associated with Norwalk-like viruses and their investigation by nested RT-PCR BMC Microbiol 1:1–14

Odelin MF et al. (2005) Investigation d'une épidémie de gastro-entérite à norovirus dans un hôpital gériatrique Ann Biol Clin (Paris) 64:141–147

Oppermann H, Mueller B, Takkinen J, Klauditz W, Schreier E, Ammon A (2001) An outbreak of viral gastroenteritis in a mother-and-child health clinic Int J Hyg Environ Health 203:369–373

Rahamat-Langendoen JC, Lokate M, Schölvinck EH, Friedrich AW, Niesters HGM (2013) Rapid detection of a norovirus pseudo-outbreak by using real-time sequence based information J Clin Virol 58:245–248

Sauvan V, Rameau YR, Pagani L, Pittet D, Harbarth S (2013) P238: Surveillance in geriatrics: what for? Which indicators? Antimicrob Resist Infect Control 2:P238

Schimmelpfennig M, Walter-Dohmes M, Aue T, Kassel G, Uphoff H, Fitzenberger J, Hauri AM (2009) [Noroviruses: on a foodborne outbreak among customers of a public canteen]. Noroviren: Zu einem lebensmittelbedingten Ausbruch unter Besuchern einer öffentlichen Kantine Epidemiologisches Bulletin 9:80–81

Schmid D et al. (2011) Foodborne gastroenteritis outbreak in an Austrian healthcare facility caused by asymptomatic, norovirus-excreting kitchen staff J Hosp Infect 77:237–241

Schmid D, Lederer I, Pichler A-M, Berghold C, Schreier E, Allerberger F (2005) An outbreak of Norovirus infection affecting an Austrian nursing home and a hospital Wien Klin Wochenschr 117:802–808

Schulz-Stübner S, Reska M, Hauer T, Schaumann R (2016) [First Results of the German consulting center for infection control outbreak registry]. Infektionen und Kolonisationen: Was können wir verbessern? Erste Ergebnisse aus dem Ausbruchsregister beim Deutschen Beratungszentrum für Hygiene Dtsch Med Wochenschr 141:e47–e52

Schwartz S et al. (2011) Norovirus gastroenteritis causes severe and lethal complications after chemotherapy and hematopoietic stem cell transplantation Blood 117:5850–5856

Showell D, Sundkvist T, Reacher M, Gray J (2007) Norovirus outbreak associated with canteen salad in Suffolk, United Kingdom Euro Surveill 12:3323

Simon A et al. (2006) Norovirus outbreak in a pediatric oncology unit Scand J Gastroenterol 41:693–699

Sinn G (2008) [On a norovirus outbreak after a restaurant visit]. Zu einem Norovirus-Ausbruch nach einem Restaurantbesuch Epidemiologisches Bulletin 6:46–47

Smith AJ et al. (2012) A large foodborne outbreak of norovirus in diners at a restaurant in England between January and February 2009 Epidemiol Infect 140:1695–1701

Sommer C, Mueller W, Resch B (2009) Two nosocomial norovirus outbreaks in the neonatal intensive and intermediate care unit Eur J Clin Microbiol Infect Dis 28:1133–1136

Sukhrie FHA, Teunis P, Vennema H, Copra C, Thijs Beersma MFC, Bogerman J, Koopmans M (2012) Nosocomial Transmission of Norovirus Is Mainly Caused by Symptomatic Cases Clin Infect Dis 54:931–937

Teunis PFM, Sukhrie FHA, Vennema H, Bogerman J, Beersma MFC, Koopmans MPG (2015) Shedding of norovirus in symptomatic and asymptomatic infections Epidem Infect 143:1710-1717

Vardy J, Love AJ, Dignon N (2007) Outbreak of acute gastroenteritis among emergency department staff Emerg Med J 24:699–702

Verbelen V et al. (2004) Hospital outbreak of gastroenteritis due to Norovirus in Belgium Acta Clin Belg 59:30–33

Vivancos R, Sundkvist T, Barker D, Burton J, Nair P (2010) Effect of exclusion policy on the control of outbreaks of suspected viral gastroenteritis: Analysis of outbreak investigations in care homes Am J Infect Control 38:139–143

Vo TH et al. (2016) An Outbreak of Norovirus Infections Among Lunch Customers at a Restaurant, Tampere, Finland, 2015 Food Environ Virol 8:174–179 Wadl M et al. (2010) Food-borne norovirus-outbreak at a military base, Germany, 2009 BMC Infect Dis 10:30

Wit de MAS, Widdowson MA, Vennema H, de Bruin E, Fernandes T, Koopmans M (2007) Large outbreak of norovirus: The baker who should have known better J Infect 55:188–193

Zingg W, Colombo C, Jucker T, Bossart W, Ruef C (2005) Impact of an Outbreak of Norovirus Infection on Hospital Resources Infect Control Hosp Epidemiol 26:263–267

Zomer TP et al. (2010) A foodborne norovirus outbreak at a manufacturing company Epidemiol Infect 138:501–506

Zühl J (2006) [Enhanced infection surveillance during the football world cup]. Erweiterte Infektionssurveillance im Rahmen der Fußballweltmeisterschaft (2) Epidemiologisches Bulletin 25:195
